# Prevalence of Coagulation Factors Deficiency among Young Adults in Saudi Arabia: A National Survey

**DOI:** 10.1055/s-0040-1721500

**Published:** 2020-12-23

**Authors:** Khalid A. AlSaleh, Nouf Al-Numair, Ayman AlSuliman, Mohammed Zolaly, Abdul Majeed Albanyan, Nouf AlOtaishan, Esra Abudouleh, Nervana Bayoumy, Ahmad Tarawah, Faisal AlZahrani, Faisal AlAllaf, Abdul kareem AlMomen, Raihan Sajid, Tarek M. Owaidah

**Affiliations:** 1 Department of Medicine, King Saud University Medical City, King Saud University, Riyadh, Saudi Arabia; 2 Department of Genetics, King Faisal Specialist Hospital and Research Centre, and College of Medicine, Alfaisal University, Riyadh, Saudi Arabia; 3 Research Center, King Faisal Specialist Hospital, Alfaisal University, Riyadh, Saudi Arabia; 4 Department of Pediatric, Taibah University, Medina, Saudi Arabia; 5 College of Medical Applied Sciences, King Saud University, Riyadh, Saudi Arabia; 6 Department of Physiology, College of Medicine, King Saud University, Riyadh, Saudi Arabia; 7 Medina Maternity and Children Hospital, Medina, Saudi Arabia; 8 Department of Pathology, Imam Abdulrahman Bin Faisal University, Dammam, Saudi Arabia; 9 Department of Molecular Medicine, Umm Al Qura University, Makkah, Saudi Arabia; 10 Department of Pathology, College of Medicine, King Saud University, Riyadh, Saudi Arabia; 11 Department of Pathology, Alfaisal University, Riyadh, Saudi Arabia; 12 Department of Pathology and Laboratory Medicine, King Faisal Specialist Hospital and Research Centre, Alfaisal University, Riyadh, Saudi Arabia

**Keywords:** inherited, bleeding, coagulation

## Abstract

**Introduction** Inherited bleeding disorders vary in prevalence due to genetic disparity and ethnicity. Little is known about the prevalence of coagulation factor deficiency and bleeding disorders in middle-eastern population.

**Methods** Young Saudi adults with at least one positive bleeding symptom reported in semi-structured validated condensed MCMDM-1vWD questionnaire were tested for complete blood count, routine and special coagulation tests, serum ferritin level, and capillary zone electrophoresis. After initial testing, those with prolonged prothrombin time (PT) or activated prothrombin time (APTT) had further testing to evaluate coagulation factors level. Platelet function was tested through platelet function analyzer (PFA)-100, and multiplate aggregometer (MEA) on patients suspected of having platelet disorders.

**Results** Six-hundred-forty patients (male = 347, 54.2%) were included. A possible platelet function defect was diagnosed in three patients with one matching Glanzmann's thrombasthenia trait pattern, and one that of Bernard-Soulier trait pattern. One patient was diagnosed with von Willebrand disease. Deficiencies in coagulation factor levels were revealed as F-VIII in 14 (7.4%), F-IX in 15 (7.6%), F-II in two (3.3%), F-V in 17 (26.1%), FVII in two (3.1%), and F-X in one (1.8%) of study subjects; low vWF activity (<50%) was found in 14 (8%). Abnormal values were found for various laboratory tests with prolongation of platelet function analyzer-epinephrine (PFA-EPI) in 11%, PFA-ADP or arachidonic acid in 15.2%, PT in 35.9%, and APTT in 63.7%. Five-hundred-seventy-six patients (90%) had normal results in the coagulation factor assays and were categorized as patients with bleeding of unknown cause (BUC). A diagnosis of a bleeding disorder was more frequently made in men than in women (38 vs. 26). Iron deficiency anemia was found in 18 (25%) females positively associated with F-IX deficiency ( *p* -value 0.000). Male gender (73.3%, *p* = 0.007) was independently associated with the diagnosis of coagulation factor deficiency.

**Conclusion** The current study reports a higher prevalence of coagulation factors deficiency in Saudi population than reported in the western population.

## Introduction

Hereditary bleeding disorders are a vast group of hemostasis abnormalities resulting from problems in platelet number, adhesion, or aggregation; or deficiencies of coagulation factors, contact factors, fibrinogen (FIB), or connective tissue. Bleeding disorders are manifested as repeated provoked or spontaneous bleeding into joints, muscles, or mucocutaneous tissues depending upon the disease severity in each individual. 1 The availability of advanced diagnostic and highly effective preventive and therapeutic measures may help maximize the benefits and minimize the risks of these disorders in diagnosing earlier than the development of complications. 2 However, quantification of bleeding disorders based upon symptoms is a challenging task. Besides severe bleeding disorders, correct diagnosis is also necessary to decide on preventive measures for mild to moderate bleeding disorders (MBDs) including platelet function disorders, von Willebrand disease (vWD), and clotting factor deficiencies. 3 Although MBDs do not lead to higher mortality rates, yet they present a potential motive for the intervention of hemostatic agents and blood products during and after surgical procedures to control bleeding or mild chronic bleeding. 4 MBDs often do not distinctively manifest earlier in life, and may become evident only after significant hemostatic challenges. Diagnostic criteria are more inconsistent for MBDs than severe bleeding disorders and have not been addressed adequately. 5 Bleeding of unknown cause (BUC), on the other hand, is a condition where MBDs cannot be associated with any genetic or hemostatic abnormality even after extensive investigation with contemporary techniques. 6

Several studies reported prevalence of inherited bleeding disorders on a small scale in Saudi population; however, objective and focus of these studies revolved around vWD, hemophilia A, hemophilia B, and platelet disorders. 7 8 Identification of cases based upon minimal symptoms may lead to overestimation of the prevalence of specific disease in population screening process. More stringent criteria in terms of accuracy and precision can be proposed to lessen the burden of a false-positive diagnosis.

Prothrombin time (PT) and activated prothrombin time (APTT) are the routinely performed coagulation tests with limited sensitivity to detect coagulation factor bleeding disorders. Platelet function analyzer (PFA)-100 is used for screening of primary hemostasis disorders with excellent sensitivity, yet its lack of specificity and predictive power of any particular disorder hampers its clinical utility as the sole testing system. Nonetheless, being a platelet functional test, it's a worthwhile addition when used in conjugation with other diagnostic and monitoring. 9 10 11

The current study aimed to link symptoms-based evaluation with routine laboratory screening testing, complete blood picture with coagulation factors, and platelet aggregation studies to predict the prevalence of coagulation factor deficiencies and platelet disorder as a cause of bleeding symptoms.

## Methods

### Study Design

Our study is based on a large national epidemiological survey which we conducted in four major regions of KSA, and we are generating several substudies with different laboratory tests conducted as part of that survey. After the multicenter IRB approval, participants from both genders were selected randomly, and only young Saudi adults were included. A semi-structured validated condensed MCMDM-1vWD Bleeding Questionnaire, including questions related to different bleeding symptoms, was used. The results of that questionnaire are presented in another under-publication manuscript. Only those participants giving a positive response to any primary question were further sampled for various blood coagulation, platelet functions testing, ferritin, and hemoglobinopathy tests. The sample collection was conducted in all four regions, but all testing was performed at Center of Excellence Thrombosis and Hemostasis, King Saud University Medical City, Riyadh—except for PFA-100 (Siemens Healthcare Diagnostics, Malvern, Pennsylvania, United States) which was performed on site.

### Sample Collection

Each of these participants had 10 mL of ethylenediaminetetraacetic acid (EDTA), 10 mL of citrated blood (at 3.2%), and 5 mL of sodium heparin was collected. Complete blood count (CBC), serum ferritin level, and capillary zone electrophoresis were performed for each participant. CBC was tested on the same day from the EDTA samples using an automated SYSMEX XN-10 instrument (Sysmex Corporation, Kobe, Japan). Serum ferritin level was measured using an automated chemistry analyzer COBAS 601 (Roche Diagnostics, Basel, Switzerland). ABO blood grouping was performed via Diamed Gel card (Changsha Yingtai Instrument Co., Ltd).

All samples for coagulation tests including PT and APTT and other coagulation factors were separated within 2 to 4 hours from collection. These samples were centrifuged and plasma separated by standard techniques which were then transported in a frozen state to the central laboratory for testing (Center of Thrombosis and Hemostasis, King Saud University Medical City, Riyadh).

To maximize the study efficiency, a group of hematologists who are part of this study designed a stepwise approach based upon initial screening tests, including PT, APTT, and PFA-100. Those with prolonged PT alone were tested for extrinsic pathways (F-II, F-V, F-VII, and F-X), and those with prolonged APTT alone were tested for intrinsic pathways (F-XI, F-X, F-XI, and F-VIII), while those who were prolonged for APTT and PFA-100 were tested for vWF Ag and function. Participants who tested normal for APTT and prolonged for PFA-100 were tested for platelet aggregation. All coagulation tests were performed on STA R Max (Diagnostica Stago, Marseille, France). Those with possible platelet disorders were called back again seeking consent for another sample collection for platelet aggregation by multiplate aggregometer.

Since ours is a national survey consisting of different physical collection sites and because of the nature of coagulation tests, the investigators set stringent measures to ensure the quality and accuracy of the testing, and samples not meeting these measures were discarded.

## Statistical Analysis

Descriptive statistics were computed as a baseline means, standard deviations, and minimum and maximum values for continuous variables. The inherited bleeding disorders were analyzed using laboratory results, including PFA, CBC, clotting factors, and ferritin. Chi-square test was used for comparing clotting factor with ferritin and hemoglobin against platelet disorder and clotting factor to confirm the severity of the factor deficiency or functional platelet disorder, which results in iron deficiency anemia (IDA). We used the software STATA v.13.0 (Stata Corp., College Station, Texas, United States) in our analysis. A statistical significance threshold of *p* < 0.05 was adopted.

## Results

Out of 848 young Saudi adults who affirmed to have at least one bleeding symptom, we performed laboratory testing on 640, where 347 (54.2%) were males, and 293 (45.7%) were females. Mean values of laboratory tests are summarized in Table 1 . For PFA test, EPI mean value was 135 ± 35 seconds ( *n* = 534), ADP was 99.8 ± 25.8 seconds ( *n* = 429), PT was 14.3 ± 1.2 seconds ( *n* = 637), and APTT was 43.8 ± 9 seconds ( *n* = 638). Abnormal values were found for laboratory PFA-EPI in 11%, PFA-ADP in 15.2%, PT in 35.9%, and APTT in 63.7%.

**Table 1:** Basic descriptive analysis for laboratory results

Variables	*N*	Mean + SD	Min	Max	Abnormal, *n* (%)
PFA-EPI	534	135 ± 35	51	300	60 (11)
PFA-ADP	429	99.8 ± 25.8	37	300	67 (15.2)
PT	637	14.3 ± 1.2	10.7	28.2	230 (35.9)
APTT	638	43.8 ± 9	29.4	172	407/638 (63.7)
F-VIII	187	101.4 ± 48.7	30	335	14/187 (7.4)
F-IX	198	104.9 ± 37.9	30	200	15/198 (7.6)
F-XI	101	108.3 ± 44	50	200	–
F-XIII	102	112.6 ± 32.4	64	200	–
vWF Ag	164	86.0 ± 28.6	34	178	–
FVIII + vWF					1 (0.5%)
vWF Act	173	78.9 ± 26.7	35	178	14 (8)
F-II	61	93.2 ± 25.5	41	250	2 (3.3)
F-V	65	68.8 ± 41	30	361	17 (26.1)
F-VII	65	78.8 ± 41	47	361	2 (3.1)
F-X	56	78.6 ± 17.2	30	149	1 (1.8)
FIB	9	266.7 ± 64.4	201	409	–
HCT	628	41.3 ± 5.2	22.4	56.5	–
MCV	628	82.4 ± 7.4	52	107.7	–
Platelet	628	272.5 ± 83.1	26	823	–
MPV	627	9 ± 1.1	1.9	14.6	–

Abbreviations: ADP, arachidonic acid; APTT, activated prothrombin time; EPI, epinephrine; FIB, fibrinogen; PFA, platelet function analyzer; PT, prothrombin time; SD, standard deviation; vWF, von Willebrand factor; HCT, hematocrit, MCV, mean corpuscular volume; MPV, mean platelet volume.

The mean values for coagulation factor-related tests were: FIB 266.7 ± 64.4 IU/mL ( *n* = 9), F-II 93.2 ± 25.5 IU/mL ( *n* = 61), F-V 68.8 ± 41 IU/mL ( *n* = 65), F-VII 78.8 ± 41 IU/mL ( *n* = 65), F-VIII 101.4 ± 48.7 IU/mL ( *n* = 187), F-IX 104.9 ± 37.9 IU/mL ( *n* = 198), F-X 78.6 ± 17.2 IU/mL ( *n* = 56), F-XI 108.3 ± 44 IU/mL ( *n* = 101), F-XIII 112.6 ± 32.4 IU/mL ( *n* = 102). The mean values of CBC variables were HCT 41.3 ± 5.2 ( *n* = 628), MCV 82.4 ± 7.4 ( *n* = 628), platelet 272.5 ± 83.1 ( *n* = 628), and MPV 9 ± 1.1 ( *n* = 627).

**Fig. 1:**
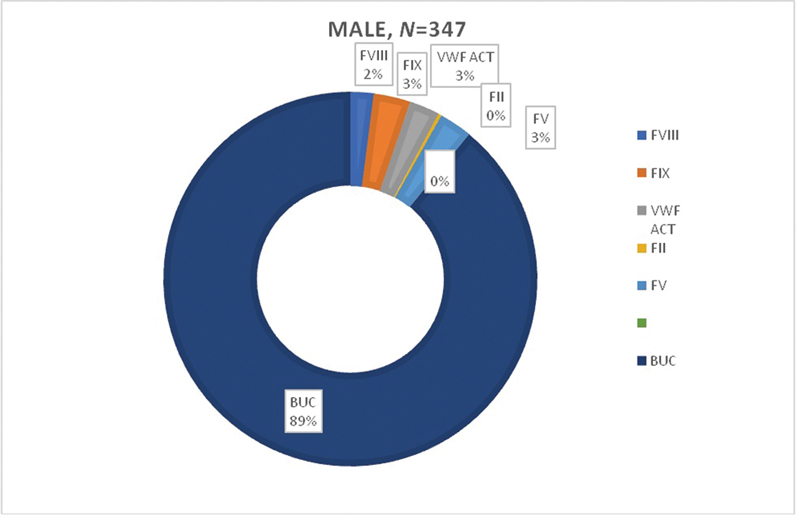
Factors deficiency in males.

**Fig. 2:**
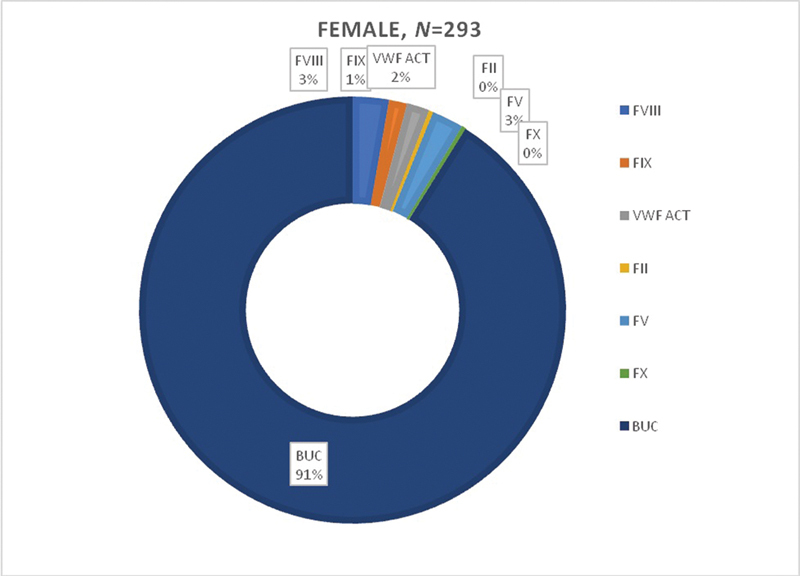
Factor deficiency in females.

We applied 50% as a cut off for the diagnosis of coagulation factor deficiency. We found low F-VIII in 7.4%, F-IX in 7.6%, vWF activity in 8%, F-II in 3.3%, F-V in 26.1%, FV-II in 3.1%, and F-X in 1.8% study subjects. Of note, among abnormal factors level, 1 (0.5%) was F-VIII + vWF Ag case ( Table 1 ). Gender-based factors deficiency is depicted in Figs. 1 and 2 , where eight females and seven males were deficient in F-VIII; four females and 11 males were deficient in F-IX ( *p* -value 0.007); one female and one male were deficient in F-II; seven females and 10 males had F-V deficiency, only one female was deficient in F-X, while five females and nine males were deficient in vWF act. The figures also show 576 (90%) participants having bleeding symptoms, but normal results in the coagulation assays and so were categorized as patients with BUC.

Platelet function tests are summarized in Table 2 : arachidonic acid (ADP) 82.8 ± 19.9 ( *n* = 26), aspirin (ASP) 91.5 ± 22.8 ( *n* = 26), collagen (COL) 74.5 ± 20.3 ( *n* = 23), ristocetin (RISTO) 86.5 ± 28 ( *n* = 26), thrombin receptor-activating peptide (TRAP) 106.1 ± 20 ( *n* = 26).

**Table 2:** Platelet function test's description

Variables	*N*	Mean + SD	Min	Max	Abnormal *n* (%)
ADP	26	82.8 ± 19.9	36	117	1 (3.8)
ASP	26	91.5 ± 22.8	46	145	6 (23.1)
COL	23	74.5 ± 20.3	40	127	5 (19.2)
RISTO	26	86.5 ± 28	42	149	7 (26.9)
TRAP	26	106.1 ± 20	65	138	6 (23.1)

Abbreviations: ADP, arachidonic acid; ASP, aspirin; COL, collagen; EPI, epinephrine; RISTO, ristocetin; SD, standard deviation; TRAP, thrombin receptor-activating peptide.

For diagnosis of platelet dysfunction, we applied the known pattern of abnormal responses to different agonists like ADP, RISTO, COL, identifying three patients in our cohort with possible platelet dysfunction (PPD), one among which matched the pattern of Glanzmann's thrombasthenia with low COL and RISTO, and one matched the pattern of Bernard Soulier with abnormal RISTO. These cases were correlated with hemoglobin and platelet count without any significant association ( Table 3 ).

**Table 3:** Comparison of PFA-EPI with PFA-ADP for normal and prolonged values

PFA-ADP	PFA-EPI normal	PFA-EPI prolonged	Total
Normal	331 (93.5%)	39 (60.9%)	370 (88.5%)
Prolonged	23 (6.5%)	25 (39.1%)	48 (11.5%)

Abbreviations: ADP, arachidonic acid; EPI, epinephrine; PFA, platelet function analyzer.

Note: *p* -Value <0.001.

For hemoglobin and ferritin, we calculated gender-based mean values. For male participants, the mean ferritin value was 97.9 ± 64.7 ( *n* = 229) while mean hemoglobin was 44.5 ± 55.8 ( *n* = 341). For female participants, the mean ferritin value was 36.4 ± 59.7 ( *n* = 110), while hemoglobin was 33.8 ± 44.8 ( *n* = 287, Table 4 ).

**Table 4:** Ferritin and Hgb per gender

Variables	*n*	Mean + SD	Min	Max	IDA
		Males			
Ferritin	229	97.9 ± 64.7	5.5	543	0
Hgb	341	44.5 ± 55.8	7.6	168	
		Females			
Ferritin	110	36.4 ± 59.7	1.65	393	18 (25%)
Hgb	287	33.8 ± 44.8	6.8	153	

Abbreviations: Hgb, hemoglobin; IDA, iron deficiency anemia.

The minimum and maximum values of variables indicate that the normal range exceeds in several cases. Therefore, we set out to analyze the proportion of test subjects with normal and abnormal ranges to identify the possible cause of bleeding. Correlation analysis between abnormal factor levels and IDA was done for both males and females. Out of male patients, low ferritin and low factor ranking was only observed in F-IX for one (2.3%).

For PFA-based tests as well as PT and APTT, values above the maximum normal ranges were considered prolonged, and they might be indicators for bleeding. Comparison between APTT and PFA-EPI and PFA-ADP is summarized in Table 4 , indicating an insignificant relationship.

## Discussion

Bleeding disorders are a cluster of inherited disorders with varying prevalence rates depending upon ethnicity. While most known inherited bleeding disorders are hemophilia A and B, they are relatively rare. 12 Most MBDs are often unrecognized as patients bleed only during stress periods, surgery, or medical procedures. 12 In Saudi Arabia, other than case reports and case series, no large scale population-based screening studies have reported the prevalence of bleeding disorders. 8 13 14 Arab population may have a higher prevalence of bleeding disorders than Western population, owing primarily to a higher consanguinity in Arab communities. 15 The goal of the current study was to conduct the first population-screening focused on bleeding disorders, correlating them with laboratory findings among young adults in Saudi Arabia.

In the current study, laboratory testing was performed for young Saudi adults who affirmed to have at least one bleeding symptom. The minimum and maximum values of variables indicated that the normal range was exceeded in several cases while ABO-corrected 16 vWFAg in four (2.4%) study subjects. Ahmed et al previously reported one factor VII deficiency, one factor X deficiency in 34 cases of inherited bleeding disorders from Eastern Province of Saudi Arabia, along with five unidentified platelet function disorders. 13 In the current study, three patients had PPD, one among which had a matching pattern of Glanzmann's thrombasthenia. 17 Al-Sharif et al reported clinical phenotype of around 20 patients with factor XIII deficiency in Riyadh region. 14 In an 8-year retrospective analysis of 168 inherited bleeding disorder patients by Al-Fawaz et al *,* patients with factor XI deficiency: four with factors V and VIII deficiency, and one with factor VII deficiency were reported, while vWD was stated as the second most common cause of hereditary bleeding disorder. 7 Sadler et al in his review, reported vWD to be one of the most common inherited bleeding diseases, with a conservative prevalence of 100 per million persons, putting the total at 580,000 persons. 18 Over the span of 25 years, Madkhali et al found 38 patients with rare clotting factor disorders; one had afibrinogenemia deficiency, two with F-II, three with F-V, six with F-VII, one with F-X, nine with F-XI, six with F-XII, and nine patients had F-XIII deficiency, while one patient had combined FV and FVIII deficiency. 19 Quiroga et al reported diagnostic efficacy of laboratory testing in vWD patients with hereditary mucocutaneous bleeding as 40.4%, and identified 17% patients with vWD, 20 while Friberg et al reported the presence of at least one bleeding symptom in 73% participants among 1,410 surveyed girls. 21 The frequency of coagulation factor abnormalities reported in the current study is greater than all these previous studies due to a larger sample size, which also indicates the greater statistical power of our study. Gebhart et al found in his hospital-based study that 25 to 50% with MBDs could be diagnosed with a specific cause, whereas in our population-based study only 10% MBDs could be diagnosed with deficiencies, remaining 90% were labeled as BUC. 6

In chi-square test of independence, a significant association was found in the current study between diagnosis and PFA-EPI and PFA-ADP results. Conflicting results have been reported previously regarding platelet function evaluated by PFA-100. A positive relationship between the extent of bleeding and platelet dysfunction was reported by Ostrowsky et al, 22 and Raman and Silverman, 23 while Fattorutto et al, 24 and Forestier et al 25 reported inconsistency in this association.

To explore the association of Hgb and ferritin with abnormalities in laboratory testing variables, the laboratory testing for coagulation factors and vWD Ag for subjects with low Hgb and abnormal Ferritin was compared. We found a correlation of abnormal ferritin level with factor X deficiency in males and with vWD Ag in females and low Hgb with FVIII abnormalities in males and females, thus, depicting the predictive nature of Hgb and ferritin for bleeding disorder identification. Low Hgb and/or ferritin are weak predictors of bleeding disorders at best, because there are various other causes of anemia and iron deficiency.

## Conclusion

The current study reports the prevalence of coagulation factor deficiency based upon never been done before population laboratory testing, which could explain the higher prevalence of bleeding disorders in our region. Coagulation factor deficiencies and vWF Ag and activity have been found associated with IDA in Saudi patients suffering from bleeding disorders. We plan on future studies based on molecular profiling.
